# Efficacy of screening with dipstick urinalysis in predicting renal function decline in healthy workers: a 10-year follow-up study

**DOI:** 10.1007/s10157-025-02703-x

**Published:** 2025-05-22

**Authors:** Machi Suka, Akira Fukui, Hiroyuki Yanagisawa

**Affiliations:** 1 Department of Public Health and Environmental Medicine, The Jikei University School of Medicine, 3-25-8 Nishi-Shimbashi, Minato-ku, Tokyo 105-8461 Japan; 2 Division of Nephrology and Hypertension, Department of Internal Medicine, The Jikei University School of Medicine, Tokyo, Japan

**Keywords:** Follow-up study, Health examination, Dipstick urinalysis, Urinary protein, Predictive accuracy, Workers

## Abstract

**Background:**

Although dipstick urinalysis has been widely used as part of health screening for employees, its efficacy in predicting renal function decline in healthy workers has not been elucidated.

**Methods:**

We conducted a 10-year follow-up study of 33,139 Japanese healthy workers to determine the association between dipstick urinalysis results (urinary protein levels) and the development of adverse kidney outcomes (rapid eGFR decline and low eGFR) and to assess the predictive accuracy of dipstick urinalysis for the 10-year incidence of adverse kidney outcomes.

**Results:**

Trace (±) and positive (1+, 2+, and 3+) dipstick proteinuria were found in 2.8% and 0.8% of the total, respectively. They had a significantly increased risk of adverse kidney outcomes than those who were negative. The sensitivity of dipstick urinalysis to discriminate between those with and without adverse kidney outcomes within 10 years was extremely low (0.01–0.07), while its specificity was nearly perfect (0.97–1.00). The sensitivity and specificity were robust to varying cutoffs (± or 1+) and to testing once or twice.

**Conclusion:**

Workers who have at least one trace or positive result on dipstick urinalysis are more likely to have declining renal function within 10 years than those who do not. The specificity of almost 1.0 suggests that this noninvasive, inexpensive test will not miss workers whose renal function is expected to decline within the next 10 years. Occupational health staff should compile a list of workers with trace and positive results and ensure that those on the list take the necessary actions, such as retests and hospital visits, in a timely manner.

## Introduction

Chronic kidney disease (CKD), which ranks among the top 10 causes of death in the United States [1] and Japan [2], is known to increase the risk of cardiovascular disease as well as progress to renal failure (end-stage kidney disease; ESKD) [3]. Although most CKD patients are asymptomatic, early detection is required [4]. Dipstick urinalysis has been used for more than 50 years as an initial screening tool to detect proteinuria, because it is noninvasive, low cost, widely available, and provides rapid results. Despite its shortcomings, [5] dipstick urinalysis applied to mass screening for public health purposes has contributed to reducing disparities in accessibility to healthcare services.

In Japan, the Occupational Health and Safety Law requires annual health examinations for employees, and one of the test items includes urinary dipstick protein. Screening for proteinuria is important for workers, because occupational exposure to toxic substances, as well as hypertension and diabetes, can cause kidney damage. However, urinary dipstick protein is often overlooked because of its low prevalence compared to hypertension, hyperglycemia, and dyslipidemia: According to the latest statistics reported by the Ministry of Health, Labour, and Welfare, the percentages of workers with hypertension, hyperglycemia, and dyslipidemia were 18.3%, 31.2%, and 13.1%, respectively, compared to 3.8% for urinary dipstick protein.[6]

The presence of proteinuria predicts future decline in renal function [7]. Several studies have been conducted on the efficacy of dipstick urinalysis in the general population, [8, 9] but no attempts to evaluate it in a working population have been reported to date. It is well known that the ‘healthy worker effect’ phenomenon is observed in studies of workers: The incidence of health problems among workers is not comparable to that of the general population, because current workers are much healthier than community residents [10]. Because of the lower incidence of health problems, the predictive ability of dipstick urinalysis for adverse kidney outcomes in healthy workers may not be necessarily equivalent to that in the general population. Nor has it been determined what the optimal cutoff of the test is for screening workers at high risk of future decline in renal function.

Using annual health examination data from 2013 to 2023, we conducted a 10-year follow-up study to determine whether dipstick urinalysis results are predictive of adverse kidney outcomes in healthy workers. Since the occurrence of ESKD is extremely rare among workers, [11] a decrease in estimated glomerular filtration rate (eGFR) was followed as a surrogate outcome [12].

## Methods

### Participants

Electronic data of the annual health examination for employees were collected from the Tokyo Health Service Association, which provides health examinations to 130,000 employees of 1400 companies in the Tokyo metropolitan area every year. Each examinee is given a unique identifier number as a means of managing his/her health examination data. The annual health examination data can be combined by a one-to-one merge based on the identifier number. The study protocol was approved by the ethics committee of the Tokyo Health Service Association (R6-6) and has been conducted in accordance with the Ethical Guidelines for Medical and Biological Research Involving Human Subjects by the Japanese Government and the Helsinki declaration.

**Fig. 1:**
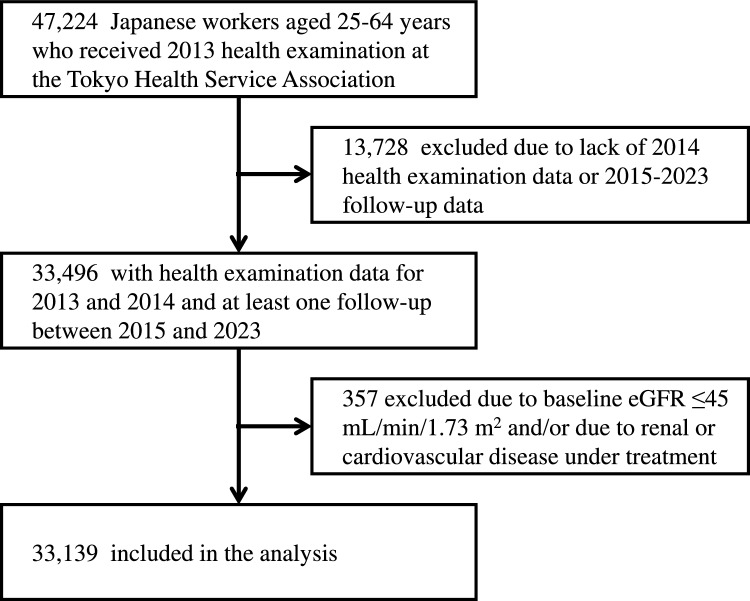
Flow diagram of sample selection

Figure 1 shows the flow diagram of sample selection. The cohort dataset for this study was constructed using annual health examination data from 2013 to 2023. Eligible participants were Japanese healthy workers aged 25–64 years with health examination data for 2013 and 2014 and at least one follow-up between 2015 and 2023. Those with an eGFR of less than 45 mL/min/1.73 m^2^ at baseline and those with any renal or cardiovascular disease under treatment were excluded. Finally, 33,139 people were included in the analysis.

### Measures

Annual health examination for employees, including anthropometric measurements, laboratory tests, and a self-administered questionnaire, was performed in compliance with the Industrial Safety and Health Act and its related regulations. Blood pressure was measured with an electronic sphygmomanometer with the participant sitting in a chair after at least 5 min of rest; if it exceeded 130/85 mmHg, blood pressure was measured again after another 5 min of rest. The lower measured value was adopted as the participant’s blood pressure. Blood (collected on an empty stomach) and urine (collected as midstream urine) samples were taken on arrival at the health examination site. All samples were immediately assayed at the laboratory of the Tokyo Health Service Association, where both internal and external quality controls of laboratory data are routinely performed in accordance with established guidelines. Dipstick proteinuria was assessed by an automated reader and recorded on five levels (–, ±, 1+, 2+, and 3+). Trace (±), 1+, 2+, and 3+ mean that the urine contains 15 mg/dL, 30 mg/dL, 100 mg/dL, and 300 mg/dL or more protein, respectively. eGFR was calculated from serum creatinine (Cr) using the equation for Japanese adults: eGFR (mL/min/1.73 m^2^)=194×Cr (mg/dl)^–1.094^×age^–0.287^×0.739 (if female).[13]

The occurrence of two changes in eGFR, ‘rapid eGFR decline’ and ‘low eGFR’, were followed as adverse kidney outcomes. ‘Rapid eGFR decline’ was defined as a consistent decline in eGFR with the rate of decline exceeding 5 mL/min/1.73 m^2^ per year in any year during the follow-up period.[14, 15] Those whose eGFR temporarily declined or fluctuated were not counted as cases. ‘Low eGFR’ was defined as eGFR falling to 45 mL/min/1.73 m^2^ (equivalent to CKD KDIGO stages G3b-5) and remaining below 45 mL/min/1.73 m^2^ thereafter.3 Those whose eGFR temporarily reached 45 mL/min/1.73 m^2^ and then improved were not counted as cases.

Hypertension was defined as systolic blood pressure ≥140 mmHg and/or diastolic blood pressure ≥90 mmHg and/or taking medication.[16] Hyperglycemia was defined as fasting blood glucose ≥126 mg/dL (7.0 mmol/L) and/or random plasma glucose ≥200 mg/dL (11.0 mmol/L) and/or hemoglobin A1c ≥6.5 % and/or taking medication.[17] Anemia was defined as hemoglobin <13 g/dL in male and <12 g/dL in female.[18]

### Statistical analysis

All statistical analyses were performed using the SAS ver. 9.4 (SAS Institute, Cary, NC, USA). Significant levels were set at *p* <0.05. Kaplan–Meier analysis with log-rank test was performed to compare the incidence of adverse kidney outcomes over 10 years. Cox proportional hazard models were used to determine the association between dipstick urinalysis results and the development of adverse kidney outcomes. Hazard ratios (HRs) with 95% confidence intervals (CIs) were calculated with adjustment for gender, age, hypertension, hyperglycemia, anemia, and eGFR at baseline. The predictive accuracy of dipstick urinalysis for the 10-year incidence of adverse kidney outcomes was assessed by sensitivity (i.e., the proportion of those with adverse kidney outcomes who are correctly identified by the test), specificity (i.e., the proportion of those without adverse kidney outcomes who are correctly excluded by the test), positive predictive value (i.e., the proportion of those with a positive test result who truly have an adverse kidney outcome), negative predictive value (i.e., the proportion of those with a negative test result who truly do not have an adverse kidney outcome), and likelihood ratios for four different combinations of cutoff (±, or 1+) and number of tests (baseline only, or baseline and the following year) [19].

## Results

Table 1 shows the baseline characteristics of the study population. Most (96.3%) of the participants were negative for dipstick proteinuria and had an eGFR of at least 60 mL/min/1.73 m^2^. Table 2 shows the comparison of baseline characteristics by urinary protein level. Due to the large sample size, statistically significant differences (*p*<0.05) were observed for most of the items listed in the table. Those who were overweight (BMI ≥25.0), had hypertension, diabetes, anemia, or smoked tended to have higher urinary protein levels.

**Table 1:** Baseline characteristics of the study population (Japanese workers aged 25–64 years, *n*=33,139)

		N	
Gender	Men	22,517	67.9%
	Women	10,622	32.1%
Age, years	<40	12,467	37.6%
	40≤	20,672	62.4%
Body mass index	≤18.5	2,724	8.2%
	18.6–24.9	22,644	68.3%
	25.0≤	7771	23.4%
Hypertension		4946	14.9%
Hypertension level		3160	9.5%
Antihypertensive medication		2587	7.8%
Diabetes		1345	4.1%
Diabetes level		1249	3.8%
Antidiabetic medication		745	2.2%
Anemia		1979	6.0%
eGFR, mL/min/1.73 m^2^	60≤	31,890	96.2%
	45–59	1249	3.8%
Urinary protein	‒	31,925	96.3%
	±	942	2.8%
	1+	171	0.5%
	2+≤	101	0.3%
Smoking habits	Non-smoker	15,889	47.9%
	Ex-smoker	4218	12.7%
	Smoker	6819	20.6%
	Unknown	6213	18.7%

Hypertension was defined as having a hypertension level (systolic blood pressure ≥140 mmHg and/or diastolic blood pressure ≥90 mmHg) and/or taking antihypertensive medication. Diabetes was defined as having a diabetes level (fasting blood glucose ≥126 mg/dL (7.0 mmol/L) and/or random plasma glucose ≥200 mg/dL (11.0 mmol/L) and/or hemoglobin A1c ≥6.5 %) and/or taking antidiabetic medication. Anemia was defined as hemoglobin <13 g/dL in men and <12 g/dL in women.

*eGFR* estimated glomerular filtration rate

**Table 2:** Comparison of baseline characteristics by urinary protein level

Urinary protein		‒ N		± N		1+ N		2+≤ N		*p*
Gender	Men	21,707	96.4%	620	2.8%	114	0.5%	76	0.3%	0.204
	Women	10,218	96.2%	322	3.0%	57	0.5%	25	0.2%	
Age, years	<40	12,046	96.6%	340	2.7%	48	0.4%	33	0.3%	0.032
	40≤	19,879	96.2%	602	2.9%	123	0.6%	68	0.3%	
Body mass index	≤18.5	2,626	96.4%	77	2.8%	14	0.5%	7	0.3%	<0.001
	18.6–24.9	21,996	97.1%	521	2.3%	87	0.4%	40	0.2%	
	25.0≤	7303	94.0%	344	4.4%	70	0.9%	54	0.7%	
Hypertension	No	27,368	97.1%	676	2.4%	95	0.3%	54	0.2%	<0.001
	Yes	4557	92.1%	266	5.4%	76	1.5%	47	1.0%	
Hypertension level	No	29,008	96.8%	776	2.6%	127	0.4%	68	0.2%	<0.001
	Yes	2917	92.3%	166	5.3%	44	1.4%	33	1.0%	
Antihypertensive medication	No	29,589	96.8%	771	2.5%	122	0.4%	70	0.2%	<0.001
	Yes	2336	90.3%	171	6.6%	49	1.9%	31	1.2%	
Diabetes	No	30,758	96.7%	817	2.6%	143	0.4%	76	0.2%	<0.001
	Yes	1167	86.8%	125	9.3%	28	2.1%	25	1.9%	
Hypertension level	No	30,846	96.7%	823	2.6%	144	0.5%	77	0.2%	<0.001
	Yes	1079	86.4%	119	9.5%	27	2.2%	24	1.9%	
Antihypertensive medication	No	31,303	96.6%	857	2.6%	154	0.5%	80	0.2%	<0.001
	Yes	622	83.5%	85	11.4%	17	2.3%	21	2.8%	
Anemia	No	30,045	96.4%	875	2.8%	151	0.5%	89	0.3%	<0.001
	Yes	1880	95.0%	67	3.4%	20	1.0%	12	0.6%	
eGFR, mL/min/1.73 m^2^	60≤	30,779	96.5%	873	2.7%	151	0.5%	87	0.3%	<0.001
	45–59	1146	91.8%	69	5.5%	20	1.6%	14	1.1%	
Smoking habits	Non-smoker	15,325	96.5%	450	2.8%	80	0.5%	34	0.2%	<0.001
	Ex-smoker	4075	96.6%	104	2.5%	27	0.6%	12	0.3%	
	Smoker	6386	93.7%	254	3.7%	40	0.6%	39	0.6%	
	Unknown	6039	97.2%	134	2.2%	24	0.4%	16	0.3%	

Hypertension was defined as having a hypertension level (systolic blood pressure ≥140 mmHg and/or diastolic blood pressure ≥90 mmHg) and/or taking antihypertensive medication. Diabetes was defined as having a diabetes level (fasting blood glucose ≥126 mg/dL (7.0 mmol/L) and/or random plasma glucose ≥200 mg/dL (11.0 mmol/L) and/or hemoglobin A1c ≥6.5 %) and/or taking antidiabetic medication. Anemia was defined as hemoglobin <13 g/dL in men and <12 g/dL in women.

*eGFR* estimated glomerular filtration rate

**Fig. 2:**
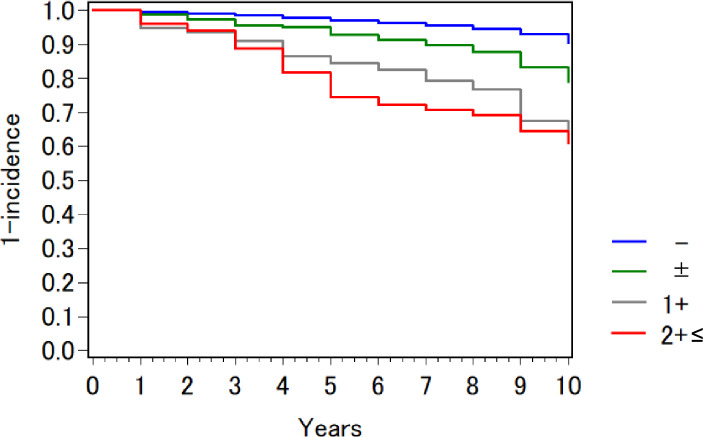
Kaplan–Meier curves for incidence of adverse kidney outcomes by urinary protein level at baseline. Participants were divided into four groups according to the dipstick urinalysis result at baseline (–, ±, 1+, and 2+≤). Adverse kidney outcomes were rapid eGFR decline (defined as eGFR slope <− 5 mL/min/1.73 m^2^ per year) and low eGFR (defined as eGFR <45 mL/min/1.73 m^2^). eGFR: estimated glomerular filtration rate

During the follow-up period, rapid eGFR decline was observed in 2,059 people (6.2%) and low eGFR in 214 people (0.6%), with 190 people (0.6%) developing both. The Kaplan–Meier analysis (Fig. 2) showed that the incidence of adverse kidney outcomes increased significantly with higher urinary protein levels (*p*<0.001). Similar results were obtained when the two adverse kidney outcomes were analyzed separately. As shown in Table 3 (a), after adjusting for plausible confounders, higher levels of urinary protein at baseline were more strongly associated with the development of adverse kidney outcomes. This association was more pronounced for low eGFR than for rapid eGFR decline.

**Table 3:** Hazard ratios for the development of adverse kidney outcomes over 10 years (Japanese workers aged 25–64 years, *n*=33,139)

	Rapid eGFR decline or low eGFR		Rapid eGFR decline		Low eGFR	
	Incidence/Total	HR (95%CI)	Incidence/Total	HR (95%CI)	Incidence/Total	HR (95%CI)
(a) Dipstick urinalysis result at baseline						
–	1,897/31,925	1.00 (reference)	1,877/31,925	1.00 (reference)	1,607/31,925	1.00 (reference)
±	116/942	1.77 (1.45–2.13)	114/942	1.81 (1.48–2.18)	26/942	2.94 (1.86–4.47)
1+	38/171	2.77 (1.97–3.77)	36/171	2.67 (1.88–3.66)	10/171	5.99 (2.94–10.86)
2+≤	32/101	3.97 (2.73–5.54)	32/101	4.52 (3.11–6.31)	18/101	19.08 (11.14–30.81)
(b) Dipstick urinalysis results at baseline and the following year						
Persistent negative (‒→‒)	1,823/31,188	1.00 (reference)	1,805/31,188	1.00 (reference)	145/31,188	1.00 (reference)
Transient trace (± → ‒ or ‒ → ±)	121/1,282	1.71 (1.41–2.05)	118/1282	1.70 (1.40–2.04)	23/1282	3.41 (2.13–5.22)
Transient positive (1+≤ → ‒ or ‒ → 1+≤)	26/224	1.77 (1.17–2.55)	25/224	1.72 (1.12–2.49)	5/224	2.76 (0.96–6.20)
Persistent trace (± → ±)	32/215	1.41 (0.97–1.98)	31/215	1.47 (1.00–2.06)	9/215	2.44 (1.13–4.62)
Positive to trace (1+≤ → ±)	12/60	2.11 (1.13–3.56)	12/60	2.29 (1.22–3.86)	4/60	5.14 (1.57–12.34)
Trace to positive (± → 1+≤)	25/78	3.05 (2.00–4.45)	25/78	(2.21–4.93)	7/78	5.02 (2.09–10.17)
Persistent positive (1+≤ → 1+≤)	44/92	6.01 (4.36–8.06)	43/92	6.56 (4.74–8.80)	21/92	25.52 (15.30–40.64)

HRs and 95% CIs were calculated with a Cox proportional hazard model adjusted for gender, age, hypertension, diabetes, anemia, and eGFR at baseline. Rapid eGFR decline was defined as eGFR slope <− 5 mL/min/1.73 m^2^ per year. Low eGFR was defined as eGFR <45 mL/min/1.73 m^2^.

*eGFR* estimated glomerular filtration rate, *HR* hazard ratio, *CI* confidence interval

**Fig. 3:**
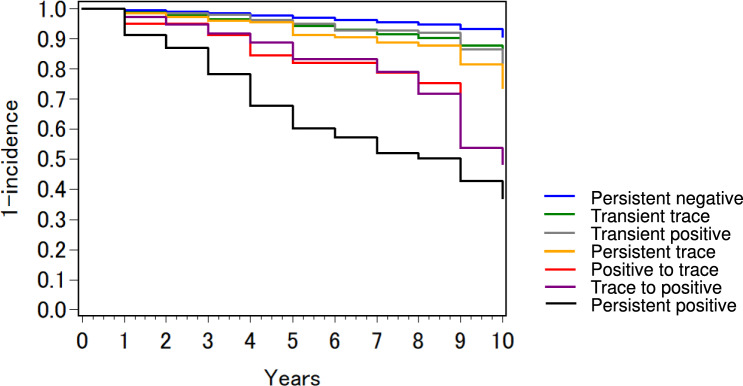
Kaplan–Meier curves for incidence of adverse kidney outcomes by urinary protein level at baseline and the following year. Participants were divided into six groups according to a combination of dipstick urinalysis results at baseline and the following year (persistent negative, transient trace, transient positive, persistent trace, positive to trace, trace to positive, and persistent positive). Adverse kidney outcomes were rapid eGFR decline (defined as eGFR slope <− 5 mL/min/1.73 m^2^ per year) and low eGFR (defined as eGFR <45 mL/min/1.73 m^2^). eGFR: estimated glomerular filtration rate.

Dipstick urinalysis results can be affected by the concentration and pH of the urine[20]. In fact, the urinary protein levels at baseline changed in the following year’s health examination for many participants. Of the 942 people who were trace (±) at baseline, 649 (68.9%) turned negative at the following year. Of the 271 people who were positive (1+, 2+, and 3+) at baseline, 120 (44.1%) and 60 (22.1%) turned negative and trace (±), respectively at the following year. Participants were divided into six groups according to a combination of dipstick urinalysis results at baseline and the following year (persistent negative, transient trace, transient positive, persistent trace, positive to trace, trace to positive, and persistent positive), and the incidence of adverse kidney outcomes was compared between groups. The Kaplan–Meier analysis (Fig. 3) showed that the incidence of adverse kidney outcomes was higher in the four groups with at least one positive result (transient positive, positive to trace, trace to positive, and persistent positive) and highest in the persistent positive group (*p*<0.001). Similar results were obtained when the two adverse kidney outcomes were analyzed separately. As shown in Table 3 (b), after adjusting for plausible confounders, all groups with at least one trace (±) or positive result had a significantly increased risk of adverse kidney outcomes than the persistent negative group.

The predictive accuracy of dipstick urinalysis for the 10-year incidence of adverse kidney outcomes was evaluated in 14,161 people with complete 10-year follow-up data. When judged by one test result (at baseline), 442 people (3.1%) had trace (±) or greater, of which 100 (0.7%) had 1+ or greater. The sensitivity and specificity to discriminate between those with and without adverse kidney outcomes within 10 years were 0.07 (85/1,136) and 0.97 (12,668/13,025), respectively, using trace (±) as the cutoff, and were 0.02 (27/1,136) and 0.99 (12,952/13,025), respectively, using 1+ as the cutoff. The positive and negative predictive values were 0.19 (89/442) and 0.92 (12,668/13,719), respectively, using trace (±) as the cutoff, and were 0.27 (27/100) and 0.92 (12,952/14,061), respectively, using 1+ as the cutoff.

When judged by two test results (at baseline and the following year), 167 people (1.2%) were both trace (±) or greater, of which 36 (0.3%) were both 1+ or greater. The sensitivity and specificity to discriminate between those with and without adverse kidney outcomes within 10 years were 0.05 (53/1,136) and 0.99 (12,911/13,025), respectively, using trace (±) as the cutoff, and were 0.01 (17/1,136) and 1.00 (13,006/13,025), respectively, using 1+ as the cutoff. The positive and negative predictive values were 0.32 (52/167) and 0.92 (12,911/13,994), respectively, using trace (±) as the cutoff, and were 0.47 (17/36) and 0.92 (13,006/14,125), respectively, using 1+ as the cutoff. The positive likelihood ratio ranged from 2.7 (minimum) when using a single test result with a cutoff of trace (±) to 10.3 (maximum) when using two test results with a cutoff of +1. The negative likelihood ratio ranged from 0.95 (minimum) when using a single test result with a cutoff of trace (±) to 0.99 (maximum) when using two test results with a cutoff of +1.

## Discussion

Although dipstick urinalysis has long been part of the annual health examination for employees in Japan, its efficacy in predicting renal function decline in healthy workers has not been elucidated. Recently, the Japanese CKD guidelines have revised the handling of dipstick urinalysis results, recommending that even a trace (±) level of urinary protein should not be left unattended: Health examinees with a trace (±) result are advised to improve their lifestyle, and if a trace (±) or positive result is found again in the next year’s health examination, they should be encouraged to seek medical consultation [21]. However, to our knowledge, there has been no epidemiological evidence to support the validity of this approach among workers. We conducted a 10-year follow-up study of 33,139 Japanese healthy workers to determine the association between dipstick urinalysis results and the development of adverse kidney outcomes (rapid eGFR decline and low eGFR) and to assess the predictive accuracy of dipstick urinalysis for the 10-year incidence of adverse kidney outcomes.

Although only a few percent of the total had trace (±) and positive dipstick proteinuria, they had a significantly increased risk of adverse kidney outcomes than those who were negative. When analyzing the dipstick urinalysis results of two consecutive years to address possible variability in dipstick urinalysis results, the risk of adverse kidney outcomes was significantly higher in the groups with at least one trace (±) or positive result than in the persistent negative group. These results support the latest guideline recommendation on how to handle health examinees with trace (±) results [21]. Previous follow-up studies of the general population revealed that trace (±) proteinuria, as well as positive proteinuria, was significantly associated with incident cardiovascular events (heart failure, myocardial infarction, stroke, and atrial fibrillation) and all-cause mortality [22, 23]. We must alert occupational health professionals to the need to compile a list of workers with trace (±) and positive dipstick proteinuria to manage their health risks. Occupational health staff should ensure that those on the list take the necessary actions, such as retests and hospital visits, in a timely manner.

The sensitivity of dipstick urinalysis to discriminate between those with and without adverse kidney outcomes within 10 years was extremely low (0.01–0.07), while its specificity was nearly perfect (0.97–1.00). The high specificity and low sensitivity in this study support the results of previous cross-sectional studies of the general population [5, 8, 24]. The sensitivity and specificity were robust to varying cutoffs (± or 1+) and to testing once or twice, which may represent a limitation of the dipstick urinalysis itself. However, at this time, there is no noninvasive, inexpensive CKD screening tool comparable to dipstick urinalysis. A previous cost-effectiveness study indicated that mass screening with dipstick urinalysis is an acceptable population strategy for CKD detection [25]. The specificity of almost 1 suggests that the test will not miss workers whose renal function is expected to decline within the next 10 years. The positive likelihood ratio of more than 10 using two test results with a cutoff of +1 suggests that workers with two positive results will certainly experience renal function decline if not treated [19]. The warning signs of declining renal function should not be overlooked to prevent more serious consequences, such as ESKD and cardiovascular diseases.

This study is the first to report the efficacy of screening with dipstick urinalysis in predicting renal function decline in healthy workers. The results of this study indicated an optimal dipstick urinalysis cutoff for screening workers at high risk of future decline in renal function and confirmed the need to manage the health risks of workers who exceed the cutoff. On the contrary, it has the following potential limitations. First, the study participants were selected from employees who received annual health examinations at the Tokyo Health Service Association. Of these, those who never had a health examination between 2015 and 2023 were excluded from the study. The selection bias may have some influence on the results. Second, information on treatment was collected in the questionnaire, but the type of medication used was not asked. Some medications are known to affect urinary protein (such as RAS inhibitors and SGLT2 inhibitors) and eGFR (such as diuretics and NSAIDs). The results of this study may have been influenced in some way by the use of medication. Third, the effect of socioeconomic status was not adjusted for in the analysis of the association between dipstick urinalysis results and the development of adverse kidney outcomes, because this information was not collected in the annual health examination for employees. However, the majority of the study population were office workers in the Tokyo metropolitan area, who are likely to have much smaller socioeconomic disparities than the general population. Since the participants were not sampled to be representative of the Japanese working population, caution should be exercised when applying the results of this study to Japan as a whole. The incidence, prevalence, and progression of CKD vary by country and ethnicity [3]. Further studies are needed to confirm whether the findings of this study are applicable to other country populations.

## Conclusion

Screening with dipstick urinalysis provides useful information for predicting future decline in renal function in healthy workers. Those who have at least one trace (±) or positive result on dipstick urinalysis are more likely to have declining renal function within 10 years than those who do not. The specificity of almost 1 suggests that this noninvasive, inexpensive test will not miss workers whose renal function is expected to decline within the next 10 years. Occupational health staff should compile a list of workers with trace (±) and positive results and ensure that those on the list take the necessary actions, such as retests and hospital visits, in a timely manner. Dipstick urinalysis results can also be used as a trigger to initiate lifestyle modifications, work environment improvements, and control of risk factors such as hypertension and diabetes, thereby increasing the likelihood that workers will continue to work in good health.

## Data Availability

The dataset of this study will not be shared because the Ethical Guidelines prohibit researchers from providing their research data to other third-party individuals.

## Declarations

### Research involving human and animals participants

The study protocol was approved by the ethics committee of the Tokyo Health Service Association (R6-6) and has been conducted in accordance with the Ethical Guidelines for Medical and Biological Research Involving Human Subjects by the Japanese Government and the Helsinki declaration.
